# G-protein regulator LGN inhibits the activity of soluble guanylyl cyclase

**DOI:** 10.1186/1471-2210-11-S1-P15

**Published:** 2011-08-01

**Authors:** Swati Chauhan, Iraida Sharina, Emil Martin

**Affiliations:** 1 University of Texas Houston Medical School, Department of Internal Medicine, Division of Cardiology, Houston, Texas, USA

## Background

Nitric oxide binds with the heme moiety of sGC and activates sGC several hundred folds the cGMP-forming activity of the enzyme. Although nitric oxide is the major physiological regulator of sGC function, the extent of this activation is influenced by a number of cellular factors, e.g. ATP, substrate GTP, reaction products, intracellular calcium, etc. Several proteins, such as HSP70, HSP90 and CCTη, where shown to interact with sGC and modulate its function.

## Results

In this report we provide evidence that demonstrate inhibition of sGC by a LGN-dependent mechanism. LGN, a Leu-Gly-Asn repeat enriched protein, is a known guanosine diphosphate (GDP) dissociation inhibitor, which regulates the Gαi family of G proteins. Using the yeast two-hybrid screening, we identified LGN as a protein that interacts with both α1 and β1 subunits of sGC. This interaction was confirmed by co-immunoprecipitation of sGC and LGN proteins from the lysate of BE2 human neuroblastoma cell line. Transient overexpression of LGN in sGC expressing MDA468 breast cancer cell line markedly decreased the activity of sGC in cell lysates in a gene-dose dependent fashion. On the contrary, inhibition of LGN expression by targeted siRNA increased sGC activity in the cellular lysates. In vitro pull-down experiments performed with truncated versions of LGN and sGC mapped the domains required for interaction between LGN and sGC. However, when the effect of purified LGN protein on the activity of purified sGC was tested in vitro, we found no evidence of inhibition, suggesting that LGN does not directly affect sGC function. When the same experiment was performed in the lysate of COS7 cell, we observed a marked inhibition of sGC.

## Conclusion

These data suggest that LGN may function as an adaptor protein for additional cellular factors inhibiting sGC. Since LGN is a known regulator of G-protein, which associates with Gα subunit, it may function as an adaptor protein for a complex consisting of sGC, LGN and Gα, thus connecting the NO-dependent and G-protein signaling pathways.

